# Synergetic Effect of Elevated Hydrostatic Pressure, Mild Heat, and Carvacrol on Inactivation of Nontyphoidal *Salmonella* Serovars in Buffered Environment

**DOI:** 10.3390/microorganisms13030498

**Published:** 2025-02-24

**Authors:** Junice Sibley, Ranju Kafle, Shahid Chowdhury, Aliyar Cyrus Fouladkhah

**Affiliations:** 1 Public Health Microbiology Laboratory, Tennessee State University, Nashville, TN 37209, USA; 2 Public Health Microbiology Foundation^SM^, Nashville, TN 37209, USA

**Keywords:** high-pressure processing, nontyphoidal *Salmonella*, *Salmonella* Tennessee, carvacrol

## Abstract

A four-strain mixture of nontyphoidal *Salmonella* and a strain of *Salmonella* Tennessee were exposed to elevated hydrostatic pressures of 350 and 650 MPa for 0 (control), 3, 5, and 10 min at temperatures of 4.4 and 60.0 °C with and without 0.2% carvacrol. Treatments were conducted in PULSE tubes inside the chamber of the Hub880 Barocycler unit. In addition to microbial counts and for better assimilation of synergism of selected extrinsic factors of the study, linear (D-value) and non-linear (k_max_) inactivation indices were calculated. A combination of mild heat, a low concentration of carvacrol, and mild pressure resulted in >5.0 log CFU/mL reduction (*p* < 0.05) in *Salmonella* serovars, surpassing the log reductions obtained by the current high-pressure processing industry standard. *Salmonella* Tennessee and the selected strain mixture exhibited comparable (*p* ≥ 0.05) sensitivity to pressure-based treatments, with D-values (350 MPa/4.4 °C) of 9.43 and 8.22 min, respectively. These values were reduced (*p* < 0.05) to 4.37 and 4.15 min, respectively, with the addition of 0.2% carvacrol to the pressure-based treatment. The application of mild heat at 60.0 °C and a low concentration of carvacrol showed microbiologically important synergism for augmenting the decontamination efficacy of high-pressure processing against nontyphoidal *Salmonella* serovars.

## 1. Introduction

Despite being first characterized more than 100 years ago, various serovars of *Salmonella* continue to be an important public health challenge in industrialized nations and emerging economies around the world [1,2,3]. Compared to other foodborne pathogens of public health concern, the nomenclature of salmonellae is relatively complex and has been subject to changes in recent years [4]. Exposure to *Salmonelle* could cause two distinctly different diseases in humans, and thus the burden of the pathogen could be discussed under the categories of typhoidal and nontyphoidal *Salmonella* serovars [2,3]. While typhoidal *Salmonella* can cause more severe clinical manifestations, such as enteric fever and gastroenteritis, its public health burden is mostly associated with poor sanitary conditions in underdeveloped regions of the world [3]. In contrast, in industrialized countries, the vast majority of salmonellosis cases are nontyphoidal in nature [3,5]. Among various nontyphoidal serovars of the pathogen, *Salmonella* Tennessee is of epidemiological and public health significance due to its involvement in a peanut butter outbreak in the early 21st century [6] that in part led to the development of important food policies and regulations in North America, including the U.S. Food Safety Modernization Act [7].

Human cases of salmonellosis are associated with diverse vehicles including direct contact with animals [8,9] and, most commonly, backyard poultry [10]. However, the vast majority of nontyphoidal salmonellosis cases in the United States are foodborne in nature [5]. An array of primary and value-added commodities including meat and meat-containing foods, fresh produce, and processed products have been linked to nontyphoidal *Salmonella* outbreaks in recent years [11,12,13].

The application of thermal processing, introduced in the early 20th century, continues to be an important intervention for ensuring the safety of global food supplies; however, the application of thermal processing for pasteurization and commercial sterilization of food commodities could impact the nutrient composition and sensory characteristics of products [14]. As such, the application of non-thermal processing including high-pressure processing represents an industrially important alternative in the food industry [15]. Due to consumers’ acceptability of pressure-treated products, their superior nutrient content and organoleptic properties, and recent advancements in engineering and the commercial availability of high-pressure processing units, the adaptation of this technology continues to gain momentum in various sectors of the food industry [16,17].

While the application of this technology is gaining widespread popularity in food commerce, the current common industry practice is the use of pressure intensity levels as high as 600 MPa for treatments lasting around 3 min [18]. The application of pressure at lower intensity levels, if validated to be microbiologically efficacious, could mitigate the potential negative impact of this technology on nutrient content and organoleptic characteristics while reducing the cost of manufacturing and maintenance of high-pressure processing units [19,20,21]. This could be achieved using synergetic effects of mild heat, in the context of thermally assisted high-pressure processing, and/or the addition of bioactive compounds. The application of pressure-based treatment coupled with the use of antimicrobial compounds provides the additional benefit of protecting the product after processing and throughout its shelf-life [18,22].

Carvacrol is a liquid plant-based phenolic bioactive compound that could be extracted from *Origanum vulgare* (oregano), *Thymus vulgaris* (thyme), and other plants. In addition to its antibacterial, antiviral, and antifungal properties, this compound has other potential biological properties such as in vitro and in vivo antioxidant and anticarcinogenic properties [23]. This compound is approved to be used as a food additive by the U.S. Food and Drug Administration as “*Food additives permitted for direct addition to food for human consumption*”. The agency recommends the use of the compounds in “*minimum quantity required to produce … intended effect*,” and thus the use of the compound at a low concentration is in harmony with regulatory recommendations [24].

Considering the public health importance of this pathogen and considering consumers’ acceptability and industrial availability of high-pressure processing, the current study aimed to investigate the synergism of mild heat, a low concentration of carvacrol, and elevated hydrostatic pressure for the inactivation of nontyphoidal *Salmonella* serovars. Additionally, our study aimed to compare the pressure sensitivity of *Salmonella* Tennessee, an epidemiologically significant serovar of the pathogen, with a four-strain nontyphoidal *Salmonella* cocktail as validation for justifying the use of this single strain interchangeably in future public health microbiology hurdle challenge studies with a similar scope. To eliminate the confounding effect of intrinsic and extrinsic characteristics of various food products, this study was conducted in a buffered environment.

## 2. Materials and Methods

### 2.1. Bacterial Cocktail Preparation and Microbial Analyses

Four strains of nontyphoidal *Salmonella* serovars, obtained from the American Type Culture Collection (ATCC; Manassas, VA, USA), were used in this study to prepare a four-strain cocktail. These strains were ATCC 14028 (serovar Typhimurium), ATCC 6962 (serovar Newport), ATCC 13076 (serovar Enteritidis), and ATCC 8387 (serovar Montevideo). These specific strains were selected based on the preliminary trials and their epidemiological significance [25,26]. In addition to these strains, a *Salmonella* Tennessee strain (ATCC 10722) was also used as a single-strain inoculum.

The handling of these strains prior to experiments is discussed in detail in the open-accessed publications of the Public Health Microbiology Laboratory of Tennessee State University [27,28]. In short, the strains were individually preserved at −80 °C in a freezer in 80% glycerol stock and were transferred for activation into sterilized Tryptic Soy Broth (TSB; Difco, Becton Dickinson, Franklin Lakes, NJ, USA) supplement with 0.6% yeast extract (TSB + YE; Legacy Biologicals, Mt. Prospect, IL, USA). They were then incubated at 37 °C for 22–24 h. For sub-culturing from each activation tube, after incubation and homogenizing the bacterial suspension by using a high-speed vortex (Scientific Industries, Bohemia, NY, USA, Model SI-0236), a 100-microliter aliquot was transferred into 10 mL of fresh sterilized TSB + YE and again incubated at the above-mentioned temperature for 22–24 h. After sub-culturing, the bacterial suspension was again homogenized using a high-speed vortex, and a loop-full, for each strain separately, was transferred onto the surface of Tryptic Soy Agar (Hardy Diagnostics, Santa Maria, CA, USA) supplemented with 0.6% yeast extract (TSA + YE) and incubated at 37 °C for 22–24 h to obtain individual colonies. After incubation, the plates were kept at 4 °C for up to one month prior to the start of the experiments. The addition of yeast extract in this and the below-mentioned media is reported to minimize the acid stress of microbial cells during storage and propagation [25].

Two days before the experiments, one loop-full from a single colony, obtained from the above-mentioned plates, for each strain separately, was transferred into 10 mL of TSB + YE, and after incubation at 37 °C for 22–24 h, similar to the above-mentioned procedure, a 100-microliter aliquot of homogenized suspension was aseptically transferred into another 10 mL of TSB + YE for incubation at 37 °C for 22–24 h for sub-culturing. The sub-cultured suspension, for each strain separately, was then exposed to centrifugal forces to harvest and purify the cells, as explained in our previously published studies [29,30]. In short, harvesting was initiated by transferring 1000 microliters of sub-cultured homogenized bacterial suspension (for each strain separately) into sterilized 1.5 mL Eppendorf centrifuge tubes. The individual tubes were then placed into a centrifuge (Centrifuge Model 5424, Eppendorf North America, Hauppauge, NY, USA; Rotor FA-45-24-11, Serial no: 5424FG367909) for 15 min at 6000 revolutions per minute (about 3548× *g* for 88 mm rotor). After centrifugation, the supernatant consisted of sloughed bacterial cell components, growth medium, and secondary metabolites was discarded, and the bacterial pellet (for each strain separately) was re-suspended in Phosphate-Buffered Saline (PBS; VWR International, Radnor, PA, USA) using the above-mentioned high-speed vortex. The re-suspended cells were once again (for each strain separately) exposed to centrifugal forces at the above-referenced intensity and time, and once again, the supernatant was discarded. The pellets (for each strain separately) were again re-suspended in PBS, and the four strains were then combined and further serially diluted for a target bacterial population level of 7 to 8 logs CFU/mL in PBS for the trials. The same procedure was used for *Salmonella* Tennessee inoculum.

After treatment with elevated hydrostatic pressure and/or the antimicrobial, the inoculated samples were first neutralized in 5 mL of Dey/Engley neutralizing broth (D/E Broth; Difco, Becton Dickinson, Franklin Lakes, NJ, USA). The samples were transferred into an ice-water slurry immediately after removal from the processing chamber to eliminate the impact of residual heat on the heat-treated samples. The cooled and neutralized samples were then 10-fold serially diluted in 0.1% maximum recovery diluent (MRD; Difco, Becton Dickinson, Franklin Lakes, NJ, USA) and were spread-plated onto TSA + YE at 37 °C for 22–24 h. Colonies were then counted manually using a Quebec colony counter (Reichert Inc., Depew, NY, USA), based on the Bacteriological Analytical Method of the U.S. Food and Drug Administration [31].

### 2.2. The Application of Elevated Hydrostatic Pressure, Mild Heat, and the Antimicrobial

The current study utilized 0.2% carvacrol (*v*/*v*) (TCI, Portland, OR, USA) based on preliminary trials and previously published studies in the Public Health Microbiology Laboratory of Tennessee State University [32,33]. Temperatures of 4.4 and 60.0 °C were chosen as they are the temperature boundaries of the United States Department of Agriculture Food Safety Inspection Services (USDA FSIS) “Danger Zone.” Limiting the storage of perishable food commodities in this zone can help entrepreneurs avoid time–temperature abuse of their products [34]. A Hub880 Barocycler unit (Pressure BioScience Inc., South Easton, MA, USA) was used to generate elevated hydrostatic pressure of up to 650 MPa in the current study. The unit’s chamber was surrounded by a stainless-steel jacket connected to a refrigerated circulating water bath (Model 160s, VWR International, Radnor, PA, USA) for precise control and adjustment of temperature. Chamber residual air was purged prior to each analysis using a pump designed with chamber closure to ensure that all treatments are hydrostatic pressure-based interventions. The pathogen was packed in PBS inside PULSE tubes (Pressure Bioscience Inc., South Easton, MA, USA) with a capacity of 1.5 mL. Every three seconds, the pressure intensity and temperature of the unit were monitored and recorded automatically using HUB PBI Software (version 2.3.11, Pressure BioScience Inc., South Easton, MA, USA). The temperature of the chamber was monitored by two K-type thermocouples (Omega Engineering Inc., Norwalk, CT, USA) inserted inside the chamber wall, secured by thermal paste (Model 5 AS5-3.5G, Arctic Silver, Visalia, CA, USA) to ensure maximum thermal conductivity and accurate and precise temperature measurements.

### 2.3. Statistical Analysis and Design

**Figure 1:**
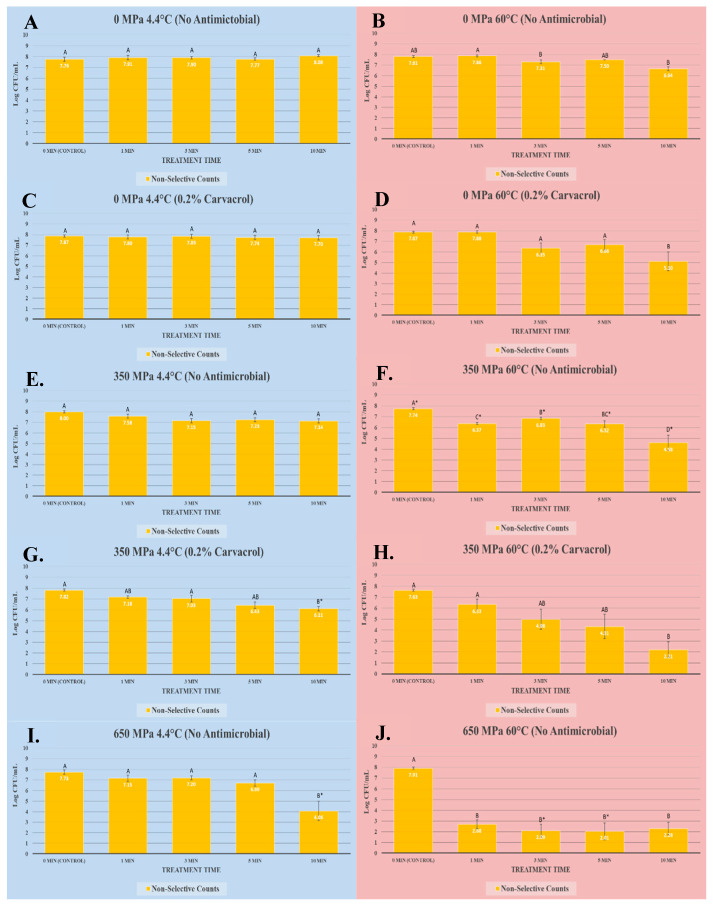
The use of elevated hydrostatic pressure, mild heat, and carvacrol for the inactivation of nontyphoidal *Salmonella* serovars.

**Figure 2:**
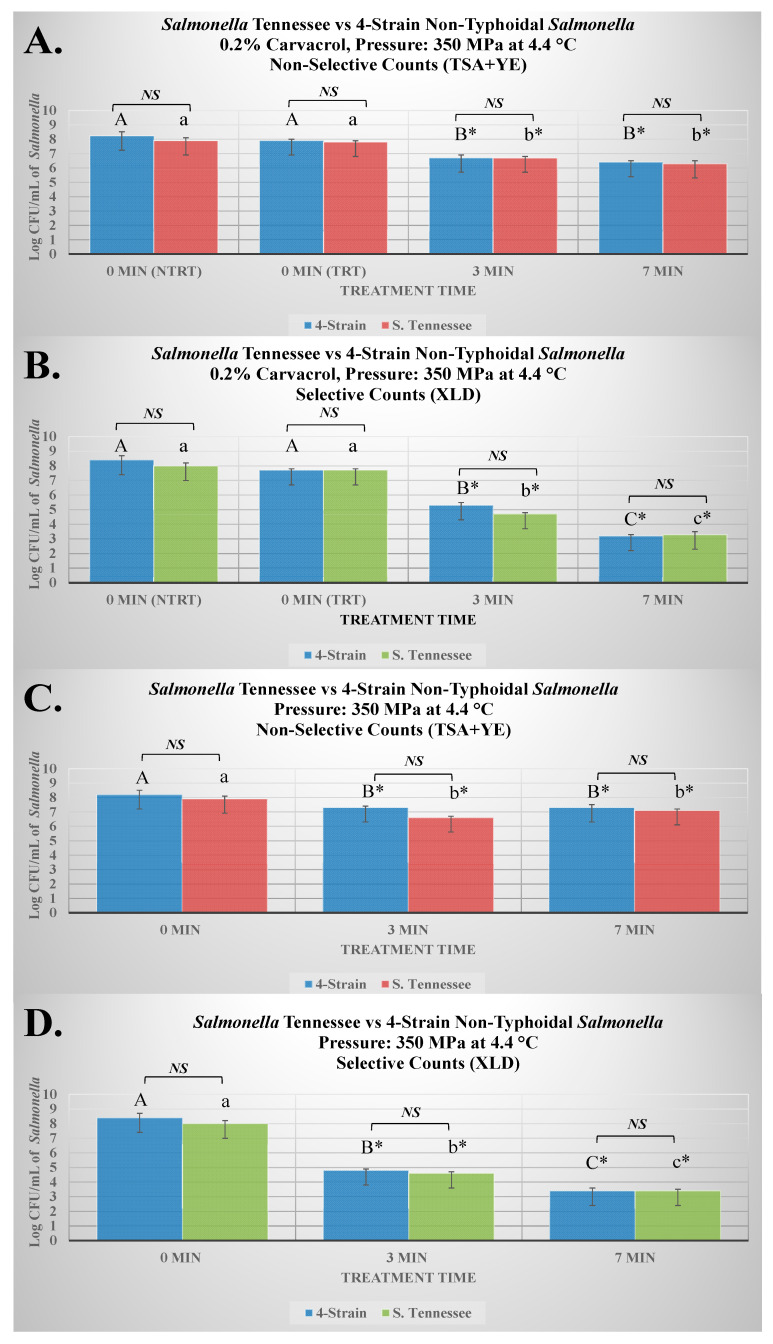
Pressure sensitivity of nontyphoidal *Salmonella* serovars and *Salmonella* Tennessee in presence and absence of carvacrol (NS = no significant difference (*p* ≥ 0.05) between *S.* Tennessee and four-strain nontyphoidal *Salmonella* serovars).

The current study was conducted using a complete randomized block design. In the study, there were two biologically independent repetitions used as blocking factors. Each block consisted of three replications, and each of these replications was additionally repeated twice, as microbiological repetitions; thus, each represented value is a mean of 12 independent observations (2 blocks with 3 replications each with 2 microbiological repetitions). The microbial counts obtained were log-transformed by using Microsoft Excel (Microsoft Corp, Redmond, WA, USA). The log-normal data were then imported to SAS_9.4_ (SAS Institute Inc., Cary, NC, USA). Initially, homogeneity of variances was checked and normality diagnostics was performed (for log-transformed counts) using the “*ods graphics*” options of the General Linear Model (GLM) procedure of SAS_9.4_. The GLM procedure was then used for conducting an Analysis of Variance (ANOVA) and two mean separation methods at a type I error level of 5%. The main analysis used was a Tukey-adjusted ANOVA which conducts all possible pair-wise comparisons of all treatments and the control(s). These analyses are showcased using letters marking each bar of the figure (Figure 1). Bars with the highest value are marked with the letter “A” and bars marked by different letters illustrate statistically significant differences (*p* < 0.05). Additionally, a Dunnett’s-adjusted ANOVA was conducted using the GLM procedure where each treatment was compared with the untreated control. This statistic is illustrated using the “*” sign on the bars in the graphs. Thus, bars marked by “*” are statistically different than the control (*p* < 0.05). Additionally, the *Proc TTEST* function of SAS_9.4_ was used to analyze the second figure (Figure 2), allowing to compare log-transformed data obtained from a single strain of *Salmonella* Tennessee with the four-strain mixture of the *Salmonella* serovars. These analyses were also conducted at the type I error level of 5%. To further exhibit the decontamination efficacy of elevated hydrostatic pressure, mild heat, and a low concentration of carvacrol, both linear (D-value) and non-linear (K_max_) inactivation indices were additionally calculated (presented in Table 1). For linear inactivation, the D-value (decimal reduction time) was calculated as the reciprocal of the slope obtained by plotting the log-transformed bacterial counts against the treatment time [35]. The non-linear inactivation indices were obtained using GInaFiT software (version 1.7, Katholieke Universiteit, Leuven, Belgium) as an add-on function to Microsoft Excel. For these analyses, the $R^{2}$ value was used as the goodness-of-fit criterion for selecting the best fitted non-linear model [36].

## 3. Results and Discussion

The current study is based on two complementary experiments. In experiment one (results presented in Figure 1), a four-strain cocktail of nontyphoidal *Salmonella* serovars was exposed to ten treatments for assimilation of the impact of elevated hydrostatic pressure and the synergism of pressure, mild heat, and a low concentration of carvacrol. These treatments (hydrostatic pressure, temperature, antimicrobial) were: (i) 0 MPa at 4.4 °C without the antimicrobial; (ii) 0 MPa at 60.0 °C without the antimicrobial; (iii) 0 MPa at 4.4 °C with 0.2% carvacrol; (iv) 0 MPa at 60.0 °C with 0.2% carvacrol; (v) 350 MPa at 4.4 °C without the antimicrobial; (vi) 350 MPa at 60.0 °C without the antimicrobial; (vii) 350 MPa at 4.4 °C with 0.2% carvacrol; (viii) 350 MPa at 60.0 °C with 0.2% carvacrol; (ix) 650 MPa at 4.4 °C without antimicrobial; (x) 650 MPa at 60.0 °C without the antimicrobial.

The second experiment (results presented in Figure 2) of the project compared the four-strain cocktail and one strain of *Salmonella* Tennessee, as an epidemiologically important serovar of public health importance. In this experiment, the two inocula were exposed to two treatments: (i) elevated hydrostatic pressure at 350 MPa at 4.4 °C without the antimicrobial and (ii) elevated hydrostatic pressure at 350 MPa at 4.4 °C with 0.2% carvacrol. The second trial was additionally plated on both non-selective (TSA + YE) and selective media (XLD, Xylose Lysine Deoxycholate).

### 3.1. Synergetic Effects of Carvacrol, Mild Heat, and Elevated Hydrostatic Pressure on Inactivation of Nontyphoidal Salmonella Serovars

Consistent with the mechanism, treatments at 0 MPa and 4.4 °C without the antimicrobial were not efficacious (*p* ≥ 0.05) in terms of leading to any reduction in nontyphoidal *Salmonella* serovars (Figure 1A). These counts (i.e., controls) were 7.76 ± 0.4, 7.91 ± 0.5, 7.90 ± 0.3, 7.77 ± 0.2, and 8.08 ± 0.2 log CFU/mL after 0, 1, 3, 5, and 10 min, respectively. Similarly, treatments at 0 MPa and at 4.4 °C with 0.2% carvacrol were not effective (*p* ≥ 0.05) in reducing the pathogen for treatments up to 10 min (Figure 1C). The pathogen counts of the control (0 treatment time) and 10 min treated samples were not statistically significant (*p* ≥ 0.05) and were 7.78 ± 0.2 and 7.70 ± 0.3 log CFU/mL, respectively (Figure 1C). This trend was also observed when samples were treated at a mild pressure of 350 MPa and 4.4 °C without the antimicrobial (Figure 1E). However, the application of mild pressure at 350 MPa in the presence of 0.2% carvacrol at 4.4 °C resulted in reductions (*p* < 0.05) in the pathogen (Figure 1G). The control count for this treatment was 7.82 ± 0.1 log CFU/mL, and after 10 min of treatment under this condition, a greater than one log (>90%) reduction (*p* < 0.05) was observed, e.g., the count of the 10 min sample was reduced (*p* < 0.05) to 6.11 ± 0.2 log CFU/mL (Figure 1G). Pathogen counts due to the synergism of mild pressure and the low concentration of the antimicrobial before 10 min were comparable to the counts obtained at a high level of elevated hydrostatic pressure without any antimicrobials. Both treatments were effective in significantly reducing (*p* < 0.05) the pathogen count at 10 min (Figure 1G,I). In other words, while 0, 1, 3, and 5 min of treatment at 650 MPa and 4.4 °C without the antimicrobial were not efficacious (*p* ≥ 0.05) in terms of leading to any reduction in the pathogen, treatment for 10 min resulted in a significant (*p* < 0.05) reduction (Figure 1I).

The mild temperature of 60.0 °C was able to greatly augment the decontamination efficacy of the treatment (Figure 1). At 0 MPa and 60.0 °C without the antimicrobial, the treatment was able to reduce (*p* < 0.05) the pathogen counts after 10 min (Figure 1B). The pathogen counts of the control (0 treatment time) and 10 min treated samples at this temperature were 7.81 ± 0.2 and 6.64 ± 0.4 log CFU/mL, respectively (Figure 1B). The synergism of mild heat (i.e., 60.0 °C) and 0.2% carvacrol exhibited greater efficacy in reducing (*p* < 0.05) the pathogen (Figure 1D). These counts were 7.87 ± 0.3, 7.88 ± 0.2, 6.35 ± 1.1, 6.66 ± 0.9, and 5.10 ± 1.7 log CFU/mL after 0, 1, 3, 5, and 10 min, respectively (Figure 1D).

Mild heat of 60.0 °C and mild elevated hydrostatic pressure of 350 MPa similarly showed microbiologically important synergism. The combination of these without antimicrobials resulted in reductions (*p* < 0.05) even after 1 min of treatment (Figure 1F). The pathogen loads of the samples at this pressure and temperature for the control and 1-, 3-, 5-, and 10-min treated samples were 7.74 ± 0.2, 6.37 ± 0.2, 6.83 ± 0.1, 6.32 ± 0.7, and 4.58 ± 1.4 log CFU/mL, respectively (Figure 1F). The reductions obtained at a mild pressure of 350 MPa at 60.0 °C were similar to those obtained at a high level of hydrostatic pressure of 650 MPa at 4.4 °C, indicating that the treatment with carvacrol is not only comparable but could actually be superior (Figure 1F,I).

A combination of mild heat (at 60.0 °C), a low concentration of carvacrol (0.2%), and mild pressure (350 MPa) similarly showed great efficacy in reducing the pathogen (Figure 1H), and understandably, the greatest reduction was achieved with a combination of high levels of elevated hydrostatic pressure and mild heat, resulting in a reduction of up to >5 log CFU/mL, which is equivalent to a reduction of >99.999% in *Salmonella* serovars, even after 1 min of treatment (Figure 1J).

In summary, we observed no major difference in bacterial reduction between treatments of 350 MPa/60.0 °C/with 0.2% carvacrol (Figure 1H) and treatments of 650 MPa/4.4 °C/without carvacrol (Figure 1H), suggesting that synergistic action can be an alternative to extreme high-pressure conditions. In other words, overall, these trials illustrate that a combination of mild heat and low levels of natural bioactive compounds such as carvacrol can synergistically augment the efficacy of elevated hydrostatic pressure (Figure 1). Thus, manufacturers and stakeholders using this technology could benefit from this synergistic effect to optimize their high-pressure processing treatment plans. The application of lower intensity pressure combined with mild heat and/or antimicrobials can not only optimize the costs of manufacturing but also assist in achieving a higher retention of nutrients and quality characteristics [37]. Additionally, the presence of low levels of natural bioactive compounds in pressure-treated products could provide protection for the product during its shelf-life [32,33]. The results of our study in a buffered environment are in harmony with previously conducted studies. As an example, the addition of 200 ppm of carvacrol was shown to enhance the microbial safety and quality of treated meat-based ready-to-eat products at 600 MPa and 25 °C [22]. Similarly, the addition of 0.75% carvacrol has been shown to be effective in augmenting the efficacy of high-pressure-processed ground poultry treated at 350 MPa for up to 10 min against *Salmonella* serovars and *Listeria monocytogenes* [38].

The success of combining various “hurdles”, such as mild heat, elevated hydrostatic pressure, and natural bioactive compounds, could be better assimilated in the context of “hurdle technology” [39,40,41]. Hurdle technology proposes the application of several antimicrobial treatments at mild levels to replace the application of one antimicrobial hurdle to an extreme extent. As an example, our study shows that the combined application of mild heat, 0.2% carvacrol, and a mild pressure of 350 MPa (Figure 1H) has a pathogen reduction capability comparable to treating a product at an extreme pressure of 650 MPa at cold temperatures (Figure 1I).

### 3.2. Sensitivity of Four-Strain Salmonella Serovars and Salmonella Tennessee to Elevated Hydrostatic Pressure and Carvacrol

The second experiment of the current study (Figure 2) compared the counts of a single strain of *Salmonella* with epidemiological significance with a four-strain mixture of *Salmonella* serovars. *Salmonella* Tennessee is a serovar of public health significance that has been involved in a recent multistate outbreak that in part resulted in the development of an important regulation in North America, the U.S. Food Safety Modernization Act [28]. As such, the current study compared this strain with a four-strain mixture of nontyphoidal *Salmonella* serovars to examine if the single strain could be interchangeable with the four-strain cocktail that has been utilized in previously published hurdle validation studies [25,26]. Additionally, this experiment compared the counts of selective and non-selective media in the presence and absence of 0.2% carvacrol for samples treated at 350 MPa at 4.4 °C (Figure 2). These parameters were selected based on preliminary trials and previously published studies of our group with the goal of testing parameters of significance to the food industry that best illustrate the synergism of carvacrol and elevated hydrostatic pressure.

It is important to note that the counts of selective medium are of great importance since the inoculated pathogen could multiply on the surface of such medium, whereas accidental contamination with environmental bacteria may not multiply on medium’s surface. This is of importance to ensure the internal validity of the study and to ensure that the presented values are true counts of the inoculated pathogen rather than accidental contamination [27]. However, the presence of selective (sodium deoxycholate) and differential (xylose and lysine) agents in the formulation of the medium can inhibit the multiplication of injured but viable cells, and thus the counts of the non-selective medium supplemented with 0.6% yeast extract better estimate the number of bacterial survivors of the treatment [42]. The selective medium additionally limits cellular repair and thus inhibits the reversal of sublethal injury needed for bacterial multiplication after exposure to stressors [38]. As such, non-selective counts are used to further calculate both linear and non-linear inactivation indices (Table 1).

Under the conditions of the current experiment for samples treated at 350 MPa and 4.4 °C with and without added 0.2% carvacrol, we observed that the selected single strain (*Salmonella* Tennessee) has comparable (*p* ≥ 0.05) sensitivity to treatments relative to the four-strain mixture (Figure 2A–D). As an example, the non-selective counts of *Salmonella* Tennessee and the four-strain nontyphoidal *Salmonella* serovars were 7.9 ± 0.2 and 8.2 ± 0.3 log CFU/mL, respectively, prior to treatments (untreated control) at 350 MPa and 4.4 °C with 0.2% carvacrol (Figure 2A). These counts were reduced (*p* < 0.05) to 6.7 ± 0.1 and 6.7 ± 0.2 log CFU/mL, respectively, after 3 min of exposure to the same treatments and were additionally reduced to 6.73 ± 0.2 and 6.40 ± 0.1 log CFU/mL, respectively, after 7 min (Figure 2A). Similar trends were observed for samples treated at the same pressure and temperature and without the added antimicrobial (Figure 2C). The non-selective counts of *Salmonella* Tennessee were 7.9 ± 0.3, 6.6 ± 0.3, and 7.1 ± 0.3 log CFU/mL after 0, 3, and 7 min of treatment at the aforementioned temperature and pressure intensity level, respectively (Figure 2C). The corresponding counts for the four-strain nontyphoidal *Salmonella* serovars were similar (*p* ≥ 0.05), being 8.2 ± 0.5, 7.3 ± 0.3, and 7.3 ± 0.1 log CFU/mL after 0, 3, and 7 min of treatments, respectively (Figure 2C). Overall, these results illustrate that, under the conditions of this experiment, *Salmonella* Tennessee and the four-strain nontyphoidal *Salmonella* serovars have comparable sensitivity to elevated hydrostatic pressure and the selected antimicrobial and thus could be used interchangeably in future public health microbiology validation studies with a similar scope.

As discussed earlier in this section and in harmony with the existing literature, we additionally observed that counts of the selective medium are appreciably lower than the supplemented non-selective medium used in this study. While the use of a selective medium is important to illustrate the internal validity of a study, the use of the supplemented non-selective medium has more accurate counts since it allows for the recovery of injured cells and thus has higher generalizability and higher external validity [37]. As an example, for samples treated for 7 min with the antimicrobial at 350 MPa and at 4.4 °C, the selective counts of *Salmonella* Tennessee and the four-strain nontyphoidal *Salmonella* serovars were 3.4 ± 0.1 and 3.4 ± 0.2 log CFU/mL, respectively (Figure 2D). Meanwhile, the corresponding non-selective counts for the same treatment were appreciably higher (*p* < 0.05) at 7.1 ± 0.3 and 7.3 ± 0.1 log CFU/mL, respectively (Figure 2C). Similar trends were observed for samples treated at 350 MPa and 4.4 °C with 0.2% carvacrol (Figure 2A,B). The selective counts of *Salmonella* Tennessee were 4.7 ± 0.2, 3.3 ± 0.2 log CFU/mL, respectively, for samples treated for 3 and 7 min (Figure 2B). The corresponding non-selective counts of the same inoculum were appreciably higher (*p* < 0.05) at 6.7 ± 0.1 and 6.3 ± 0.2 log CFU/mL, respectively, for the same treatments (Figure 2A).

The impact of independent variables (heat, pressure, antimicrobial) on the dependent variable (pathogen counts) could additionally be described by examining the linear and non-linear inactivation indices associated with these treatments (Table 1). As an example, the D-values (linear inactivation index) for *Salmonella* Tennessee and the four-strain nontyphoidal *Salmonella* serovars were similar, being 9.43 and 8.22 min, respectively, for samples treated at 350 MPa and 4.4 °C without carvacrol (Table 1). The corresponding counts for the samples treated in the presence of 0.2% carvacrol were 4.37 and 4.15 min (Table 1). Similar trends were observed for inactivation indices obtained using non-linear models (Table 1).

Using other processing techniques such as manothermosonication, in a recent study, it was similarly observed that a log-linear model could accurately predict the efficacy of a treatment against *Salmonella* serovars [43]. The synergistic impact we observed in this study is in harmony with the published literature. As an example, the pressure intensity of 400 MPa and various bactericidal compounds were shown to work synergistically to limit the risk of various foodborne pathogens including *Salmonella* serovars [44]. A similar conclusion was observed using 13 natural bioactive compounds to synergize the impact of high-pressure processing against both Gram-positive and Gram-negative microorganisms [45]. Even at a low elevated hydrostatic pressure of 300 MPa, other natural compounds such as vanillin also exhibited the capability to augment the decontamination efficacy of the treatment [46]. Carvacrol primarily targets the bacterial cell membrane and disrupts the membrane’s function and structure [47]. The synergistic effect of this compound with mild heat and pressure could be attributed to the fact that all three lead to the disintegration of the outer membrane of bacterial cells and, thus, have similar targets for disrupting bacterial cell activities.

## 4. Conclusions

Under the conditions of our experiments, we observed that mild heat and a low concentration of the chosen natural antimicrobial could greatly augment the decontamination capability of high-pressure processing. This synergetic effect could perhaps be described best in the context of hurdle technology where a combination of mild treatments, as hurdles, could be more beneficial for the safety and quality of a product relative to the use of one “hurdle” at an extreme extent. It is noteworthy that testing the synergism in buffered conditions enables us to test a concept rather than a specific product, potentially providing results with external validity and without confounding effects of intrinsic factor(s) of a specific commodity. As such, the application of the results of this study for any specific product requires further hurdle validation studies to ensure the safety, quality, and maintenance of organoleptic properties.

In our experiments, we additionally observed that *Salmonella* Tennessee, an epidemiologically important serovar, has comparable sensitivity to the tested four-strain mixture of nontyphoidal *Salmonella*. This illustrates that *Salmonella* Tennessee could be used interchangeably in future public health microbiology validation studies. Our study finally showed statistically, and biologically significant disparities associated with counts obtained from selective and non-selective media. The use of a selective medium is of great importance to ensure the internal validity of the study, i.e., to ensure that the counts obtained are truly associated with the pathogen inocula and not impacted by accidental laboratory cross-contamination of samples. However, we observed that selective counts are consistently lower than those obtained by non-selective counts supplemented with 0.6% yeast extract. Thus, to ensure the generalizability of a study, counts of the supplemented non-selective medium provide higher external validity.

## Figures and Tables

**Table 1:** Linear and non-linear (biphasic model) inactivation indices for reduction of nontyphoidal *Salmonella* serovars and *Salmonella* Tennessee.

		Linear Inactivation Indices	Non-Linear Inactivation Indices	
Pathogen	Treatment	D-value (min)	K_max1_ (1/min)	K_max2_ (1/min)
*Salmonella* Tennessee (one strain)	350 MPa at 4.4 °C without antimicrobial	9.43 (R^2^ = 0.26)	9.61 (R^2^ = 0.70)	0.00 (R^2^ = 0.70)
Nontyphoidal *Salmonella* (4 strains)	350 MPa at 4.4 °C without antimicrobial	8.22 (R^2^ = 0.43)	9.15 (R^2^ = 0.66)	0.00 (R^2^ = 0.66)
*Salmonella* Tennessee (one strain)	350 MPa at 4.4 °C with 0.2% Carvacrol	4.37 (R^2^ = 0.80)	1.14 (R^2^ = 0.87)	0.13 (R^2^ = 0.87)
Nontyphoidal *Salmonella* (4 strains)	350 MPa at 4.4 °C with 0.2% Carvacrol	4.15 (R^2^ = 0.72)	2.04 (R^2^ = 0.83)	0.16 (R^2^ = 0.83)

## Data Availability

The datasets of the current study can be obtained by contacting the study’s corresponding author with reasonable requests. A request could be submitted by obtaining the contact information from the Public Health Microbiology Foundation^SM^ at https://publichealthmicrobiology.education/ (accessed on 26 January 2025). The SAS codes used for statistical analyses in the current study were derived from no-cost and publicly available sources with needed modifications and can be obtained by contacting the study’s corresponding author with reasonable requests.

## References

[1] Logue C.M., De Cesare A., Tast-Lahti E., Chemaly M., Payen C., LeJeune J., Zhou K. (2024). *Salmonella* spp. in poultry production—A review of the role of interventions along the production continuum. Adv. Food Nutr. Res..

[2] Godínez-Oviedo A., Cuellar-Núñez M.L., Luzardo-Ocampo I., Campos-Vega R., Hernández-Iturriaga M. (2021). A dynamic and integrated in vitro/ex vivo gastrointestinal model for the evaluation of the probability and severity of infection in humans by *Salmonella* spp. vehiculated in different matrices. Food Microbiol..

[3] Eng S.K., Pusparajah P., Ab Mutalib N.S., Ser H.L., Chan K.G., Lee L.H. (2015). *Salmonella*: A review on pathogenesis, epidemiology and antibiotic resistance. Front. Life Sci..

[4] Grimont P.A., Weill F.X. (2007). Antigenic Formulae of the Salmonella Serovars.

[5] Scallan E., Hoekstra R.M., Angulo F.J., Tauxe R.V., Widdowson M.A., Roy S.L., Jones J.L., Griffin P.M. (2011). Foodborne illness acquired in the United States—Major pathogens. Emerg. Infect. Dis..

[6] Centers for Disease Control and Prevention (2007). Multistate outbreak of *Salmonella* serotype Tennessee infections associated with peanut butter—United States, 2006–2007. Morb. Mortal. Wkly. Rep..

[7] Fouladkhah A. (2017). The Need for evidence-based outreach in the current food safety regulatory landscape. J. Ext..

[8] Frey E., Stapleton G.S., Nichols M.C., Gollarza L.M., Birhane M., Chen J.C., McCullough A., Carleton H.A., Trees E., Hise K.B. (2024). Antimicrobial resistance in multistate outbreaks of nontyphoidal *Salmonella* infections linked to animal contact—United States, 2015–2018. J. Clin. Microbiol..

[9] Ricchi M., Filippi A., Scaltriti E., Tambassi M., Pongolini S., Bolzoni L., Prosperi A., Torreggiani C., Cammi M., Chiatante A. (2025). Outbreak of *Salmonella* enterica subsp. enterica Serovar Napoli on a Dairy Cow Farm. Animals.

[10] Stapleton G.S., Habrun C., Nemechek K., Gollarza L., Ellison Z., Tolar B., Koski L., Brandenburg J.M., Salah Z., Palacios A. (2024). Multistate outbreaks of salmonellosis linked to contact with backyard poultry—United States, 2015–2022. Zoonoses Public Health.

[11] Waltenburg M.A., Salah Z., Canning M., McCain K., Rickless D., Ablan M., Crawford T.N., Low M.S.F., Robyn M., Molinari N.A.M. (2025). Demographic Characteristics and County-level Indicators of Social Vulnerability in Salmonellosis Outbreaks Linked to Ground Beef—United States, 2012–2018. J. Food Prot..

[12] Rose-Martel M., Tamber S. (2025). Analysis of Outbreak Data Reveals Factors Contributing to Salmonellosis Outbreaks Linked to Cantaloupes. J. Food Prot..

[13] Jain S., Bidol S.A., Austin J.L., Berl E., Elson F., Williams M.L., Deasy M., Moll M.E., Rea V., Vojdani J.D. (2009). Multistate outbreak of *Salmonella* Typhimurium and Saintpaul infections associated with unpasteurized orange juice—United States, 2005. Clin. Infect. Dis..

[14] Azizi-Lalabadi M., Moghaddam N.R., Jafari S.M. (2023). Pasteurization in the food industry. Thermal Processing of Food Products by Steam and Hot Water.

[15] Tao Y., Sun D.W., Hogan E., Kelly A.L. (2014). High-pressure processing of foods: An overview. Emerging Technologies for Food Processing.

[16] Huang H.W., Wu S.J., Lu J.K., Shyu Y.T., Wang C.Y. (2017). Current status and future trends of high-pressure processing in food industry. Food Control.

[17] Nabi B.G., Mukhtar K., Arshad R.N., Radicetti E., Tedeschi P., Shahbaz M.U., Walayat N., Nawaz A., Inam-Ur-Raheem M., Aadil R.M. (2021). High-pressure processing for sustainable food supply. Sustainability.

[18] Aganovic K., Hertel C., Vogel R.F., Johne R., Schlüter O., Schwarzenbolz U., Jäger H., Holzhauser T., Bergmair J., Roth A. (2021). Aspects of high hydrostatic pressure food processing: Perspectives on technology and food safety. Compr. Rev. Food Sci. Food Saf..

[19] Rastogi N.K., Raghavarao M.S., Balasubramaniam V.M., Niranjan K., Knorr D. (2007). Opportunities and challenges in high pressure processing of foods. Crit. Rev. Food Sci. Nutr..

[20] Khouryieh H. (2024). Impact of High Pressure Processing on the Safety and Quality of Food Products: A Review. Recent Adv. Food Nutr. Agric..

[21] Queirós R.P., González-Angulo M., Tonello-Samson C. (2025). Challenges and innovations in high pressure processing commercial implementation. Innovative Food Packaging and Processing Technologies.

[22] de Oliveira T.L.C., Junior B.R.D.C.L., Ramos A.L., Ramos E.M., Piccoli R.H., Cristianini M. (2015). Phenolic carvacrol as a natural additive to improve the preservative effects of high pressure processing of low-sodium sliced vacuum-packed turkey breast ham. LWT-Food Sci. Technol..

[23] Sharifi-Rad M., Varoni E.M., Iriti M., Martorell M., Setzer W.N., del Mar Contreras M., Salehi B., Soltani-Nejad A., Rajabi S., Tajbakhsh M. (2018). Carvacrol and human health: A comprehensive review. Phytother. Res..

[24] US Food and Drug Administration Code of Federal Regulations Title 21. Part 172. https://www.accessdata.fda.gov/scripts/cdrh/cfdocs/cfcfr/cfrsearch.cfm?fr=172.515.

[25] Allison A., Daniels E., Chowdhury S., Fouladkhah A. (2018). Effects of elevated hydrostatic pressure against mesophilic background microflora and habituated *Salmonella* serovars in orange juice. Microorganisms.

[26] Allison A., Fouladkhah A.C. (2021). Sensitivity of Planktonic Cells and Biofilm of Wild-Type and Pressure-Stressed *Cronobacter sakazakii* and *Salmonella* enterica Serovars to Sodium Hypochlorite. Food Protect. Trends.

[27] Chowdhury A., Aras S., Kabir N., Wadood S., Allison A., Chowdhury S., Fouladkhah A.C. (2021). Susceptibility of pathogenic nontyphoidal *Salmonella* serovars and avirulent *Salmonella* LT2 to elevated hydrostatic pressure and citricidal^TM^. J. Tenn. Acad. Sci..

[28] Asefaw S., Aras S., Kabir M.N., Wadood S., Chowdhury S., Fouladkhah A.C. (2023). Public health importance of preventive measures for *Salmonella* Tennessee and *Salmonella* typhimurium strain LT2 biofilms. Microbiol. Res..

[29] Fouladkhah A., Geornaras I., Yang H., Belk K.E., Nightingale K.K., Woerner D.R., Smith G.C., Sofos J.N. (2012). Sensitivity of Shiga toxin-producing *Escherichia coli*, multidrug-resistant *Salmonella*, and antibiotic-susceptible *Salmonella* to lactic acid on inoculated beef trimmings. J. Food Protect..

[30] Fouladkhah A., Geornaras I., Yang H., Sofos J.N. (2013). Lactic acid resistance of Shiga toxin-producing *Escherichia coli* and multidrug-resistant and susceptible *Salmonella* Typhimurium and *Salmonella* Newport in meat homogenate. Food Microbiol..

[31] United States Food and Drug Administration (2001). Bacteriological Analytical Methods (FDA BAM). Aerobic Plate Count. https://www.fda.gov/food/laboratory-methods-food/bam-chapter-3-aerobic-plate-count.

[32] George J., Aras S., Kabir M.N., Wadood S., Chowdhury S., Fouladkhah A.C. (2020). Sensitivity of planktonic cells of *Staphylococcus aureus* to elevated hydrostatic pressure as affected by mild heat, carvacrol, nisin, and caprylic acid. Int. J. Environ. Res. Public Health.

[33] Kabir M.N., Aras S., Allison A., Adhikari J., Chowdhury S., Fouladkhah A. (2019). Interactions of carvacrol, caprylic acid, habituation, and mild heat for pressure-based inactivation of O157 and non-O157 serogroups of Shiga toxin-producing *Escherichia coli* in acidic environment. Microorganisms.

[34] United States Department of Agriculture Food Safety Inspection Service. “Danger Zone” (40°F–140°F). https://www.fsis.usda.gov/food-safety/safe-food-handling-and-preparation/food-safety-basics/danger-zone-40f-140f.

[35] Koutsoumanis K.P., Lianou A., Gougouli M. (2016). Latest developments in foodborne pathogens modeling. Curr. Opin. Food Sci..

[36] Geeraerd A.H., Valdramidis V.P., Van Impe J.F. (2005). GInaFiT, a freeware tool to assess non-log-linear microbial survivor curves. Int. J. Food Microbiol..

[37] Agregán R., Munekata P.E., Zhang W., Zhang J., Pérez-Santaescolástica C., Lorenzo J.M. (2021). High-pressure processing in inactivation of *Salmonella* spp. in food products. Trends Food Sci. Technol..

[38] Chuang S., Sheen S., Sommers C.H., Zhou S., Sheen L.Y. (2020). Survival evaluation of *Salmonella* and *Listeria monocytogenes* on selective and nonselective media in ground chicken meat subjected to high hydrostatic pressure and carvacrol. J. Food Prot..

[39] Leistner L. (2000). Basic aspects of food preservation by hurdle technology. Int. J. Food Microbiol..

[40] Singh S., Shalini R. (2016). Effect of hurdle technology in food preservation: A review. Crit. Rev. Food Sci. Nutr..

[41] Khan I., Tango C.N., Miskeen S., Lee B.H., Oh D.H. (2017). Hurdle technology: A novel approach for enhanced food quality and safety–A review. Food Control.

[42] Zhou S., Sheen S., Zhao G., Chuang S., Liu L. (2020). Prediction of *Salmonella* inactivation in sliced tomato subject to high pressure processing and trans-cinnamaldehyde treatment using selective and non-selective growth media for survival evaluations. Food Control.

[43] Beitia E., Ebert E., Plank M., Chanos P., Hertel C., Bhonsale S.S., Van Impe J.F., Heinz V., Aganovic K., Valdramidis V. (2024). Modelling of *Salmonella* Enteritidis inactivation in liquid whole egg under dynamic manothermosonication treatments. Innov. Food Sci. Emerg. Technol..

[44] Garriga M., Aymerich M.T., Costa S., Monfort J.M., Hugas M. (2002). Bactericidal synergism through bacteriocins and high pressure in a meat model system during storage. Food Microbiol..

[45] Feyaerts J., Rogiers G., Corthouts J., Michiels C.W. (2015). Thiol-reactive natural antimicrobials and high pressure treatment synergistically enhance bacterial inactivation. Innov. Food Sci. Emerg. Technol..

[46] Pina-Pérez M.C., Silva-Angulo A.B., Muguerza-Marquinez B., Aliaga D.R., López A.M. (2009). Synergistic effect of high hydrostatic pressure and natural antimicrobials on inactivation kinetics of Bacillus cereus in a liquid whole egg and skim milk mixed beverage. Foodborne Pathog. Dis..

[47] Di Pasqua R., Betts G., Hoskins N., Edwards M., Ercolini D., Mauriello G. (2007). Membrane toxicity of antimicrobial compounds from essential oils. J. Agric. Food Chem..

